# Can We Pick the Pocket of Post-Intensive Care Syndrome?

**DOI:** 10.1192/j.eurpsy.2022.1749

**Published:** 2022-09-01

**Authors:** K. Philbrick, E. Bieber, L. Karnatovskaia

**Affiliations:** 1 Mayo Clinic, Psychiatry, Rochester, United States of America; 2 Lurie Children’s Hospital, Psychiatry, Chicago, United States of America; 3 Mayo Clinic, Pulmonary & Critical Care Medicine, Rochester, United States of America

**Keywords:** post-intensive care syndrome, posttraumatic stress, psychological support by positive suggestion, ICU care

## Abstract

**Introduction:**

Post-intensive care syndrome, which includes symptoms of anxiety, depression, and posttraumatic stress, afflicts one-third of critical illness survivors. Symptoms persist and significantly degrade quality of life. No intervention has earned clear evidence of reducing these adverse psychological sequelae. Building on earlier pilot data, psychological support based on positive suggestions (PSBPS), is being investigated in an ongoing, randomized, controlled prospective trial across multiple intensive care unit (ICU) settings in a large, tertiary medical center.

**Objectives:**

Recognizing that even sedated patients perceive and internalize communication, we share lessons learned thus far in the art of engaging with sedated, often unresponsive patients.

**Methods:**

Our presentation describes this NIH-funded PSBPS study, including the preparatory training and subsequent implementation of a structured script delivered daily to ICU patients, regardless of cognitive status or ability to respond. To interfere with the initial process of fear conditioning/negative memory formation, we introduce mitigating information about potentially traumatic events during the temporal window when initial memory consolidation occurs, reframing the alien, often frightening ICU environment while providing positive suggestions of safety and healing.

**Results:**

Psychiatrists characteristically engage alert, communicative patients. Unfortunately, when meaningful cognitive exchange is impossible, further effort is often limited. By contrast, choosing to engage ventilated, sedated patients with active re-interpretation is a novel enterprise. We share technique and lessons learned from the first two years.

**Conclusions:**

Consultation psychiatrists are uniquely situated to explore with our critical care colleagues how best to mitigate the corrosive psychological consequences of intensive care and improve the future of ICU survivors.

**Disclosure:**

No significant relationships.

